# The Analysis of Pressed Cups Producing Possibilities from Rolled Bimetallic Al-1050 + Cu-M1E Sheets

**DOI:** 10.3390/ma13102413

**Published:** 2020-05-25

**Authors:** Dariusz Rydz, Grzegorz Stradomski, Arkadiusz Szarek, Katarzyna Kubik, Piotr Kordas

**Affiliations:** 1 Faculty of Production Engineering and Materials Technology, Czestochowa University of Technology, 19 Armii Krajowej Av., 42-200 Czestochowa, Poland; rydz.dariusz@wip.pcz.pl (D.R.); kubik.katarzyna@wip.pcz.pl (K.K.); piotr.kordas@pcz.pl (P.K.); 2 Faculty of Mechanical Engineering and Computer Science, Czestochowa University of Technology, 21 Armii Krajowej Av., 42-200 Czestochowa, Poland; arek@iop.pcz.pl

**Keywords:** bimetal, sheet metal forming, asymmetrical of rolling, deep drawing, sheet buckling

## Abstract

Drawability tests of metal sheets are known and used as technological processes that allow assessing possibilities of plastic forming. One such test is the cupping test, which is very useful for examining thin sheets of both uniform and multilayer materials. In this work, a comprehensive analysis of the shaping of the bimetallic product Al–Cu (Al-1050 + Cu-M1E) was carried out. The research covers the entire production cycle, from explosive-welding, through asymmetric rolling (ASR) to deep drawing. The scientific and cognitive aspect of the work is to determine the potential of plastic-forming processes without the need for interoperational heat treatments. Tests were carried out for two variants of bimetals used in tools: matrix–Al-1050 + Cu-M1E and matrix–Cu-M1E + Al-1050.

## 1. Introduction

Dynamic increase in demands in industrial conditions for bimetallic products has been recently observed. The production technology of bimetallic products consists of several processes. The first stage is to obtain a permanent connection of two metals, or alloys. The second is plastic-shaping of the combined blank [1,2,3,4,5,6,7,8,9,10]. At this stage, often several plastic work-processes are carried out, the purpose of which is to finally shape the multilayered product [11,12,13,14,15,16,17,18,19,20,21,22,23,24]. The third and final process is heat treatment. However, in the case of multilayer products, it often causes the formation of intermetallic phases (even layers), which in turn, generate microcracks or delamination of the welded materials [24,25,26,27,28,29,30,31,32,33,34]. Usually delamination of the welded materials occurs as effect of improper heat treatment [10,18,24,34].

This work contains a comprehensive consideration of the welding materials used in bimetal asymmetrical rolling and drawing of multilayer products. Despite great demands of bimetallic products, due to the difficulties during their plastic formation, their production in Poland—and even the world in general—is limited.

The limiting step for further shaping of thin sheets after the rolling process is conducting them through deep-drawing processes [5,6,7]. This was the primary reason why the process of multi-operational drawing of thin bimetallic Al-1050 + Cu-M1E sheets has been taken into consideration in this work. This study also discusses two pre-pressing processes, i.e., explosive-welding and requiring continuous control of the asymmetrical rolling process (ASR) [3,9,10,17,23,33,34].

**Figure 1:**
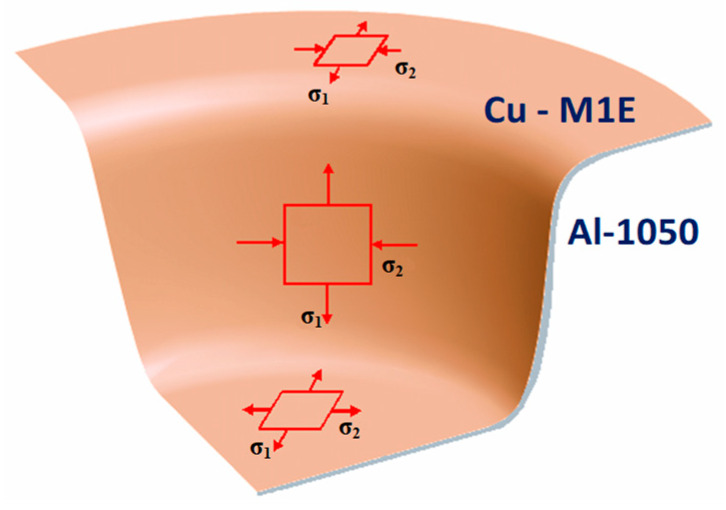
Diagram of the main stresses state during stamping of a drawpiece. Based on [2,5,6].

Deforming thin flat sheets into so-called drawpieces (i.e., with an faultless surface) takes place in the stamping process. Generally, it is assumed that a planar-stress state occurs during pressing of thin sheet metal (Figure 1). The main stress σ_3_ ≅ 0 and is negligible. In contrast, the main stresses σ_1_ and σ_2_ occurring in the sheet depend on the location during the stamping process [5,6].

For the most cases of shaping, the edge of the sheet is not restrained during the drawing process. Therefore, forming of the drawing piece takes place with a predominant proportion of drawing. A similar phenomenon occurs during the deep-drawing process the aim of which is the increase of the height of the die-stamping at the effect of its lateral dimensions.

**Figure 2:**
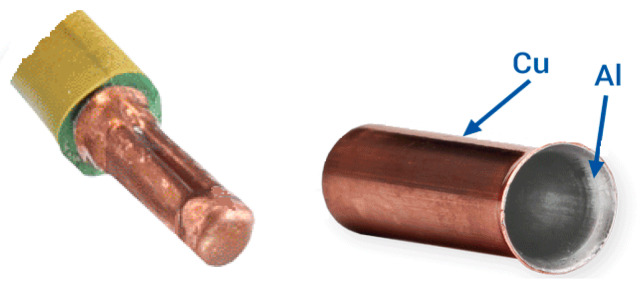
Example application of a bimetal clamp for an aluminum conductor and a bimetallic cup used for electric connectors. Based on [2].

In industrial conditions, a continuous increase of application of bimetallic products is observed. This is clearly visible in the energy and electronics sectors, where products based on aluminum and copper are more commonly used. To date, the process of shaping flat, multilayer products has been extensively described in many papers [9,10,18,34]. The combination of aluminum and copper is quite commonly used in industrial conditions [2,7,8,9]. In energy and electronics systems, metal composites in the form of aluminum-copper elements (Al/Cu) are very often used. Usually, as a result of the high-density electric current flow through such elements—as well as a result of external interactions—it heats up, which, with additional mechanical load, results in the development of rheological processes. The study of the influence of short and long-term thermal impacts on loaded structural elements made of Al/Cu bimetal is necessary to ensure their safe operation. Copper and aluminum terminals of the HMA type are one such example of their application (Figure 2). The cup presented in Figure 2 is an element applied to an aluminum cable and clamped. This gives the possibility, for example, to avoid problems with the connector sparking two different cables made of Cu and Al. Such a solution allows t reducing costs and weight of extension cords by replacing copper cables with their aluminum substitutes. In addition, the use of the HMA connector protects the aluminum conductor with copper terminal from galvanic corrosion [2]. Industry requires developing new technologies for the manufacturing of products with increasingly complex shapes, such as HMA Al–Cu cable terminals, Al–Cu bimetallic cable terminals, thermal switches, bimetallic meters, etc. One of the most important research methods underlying the development of such new technologies is the extrusion process, combined with pumping. If, in this shaping process, the material maintains the continuity of the connection and does not break after rolling, it can be concluded that other methods of plastic shaping are possible [3,5,6].

**Figure 3:**
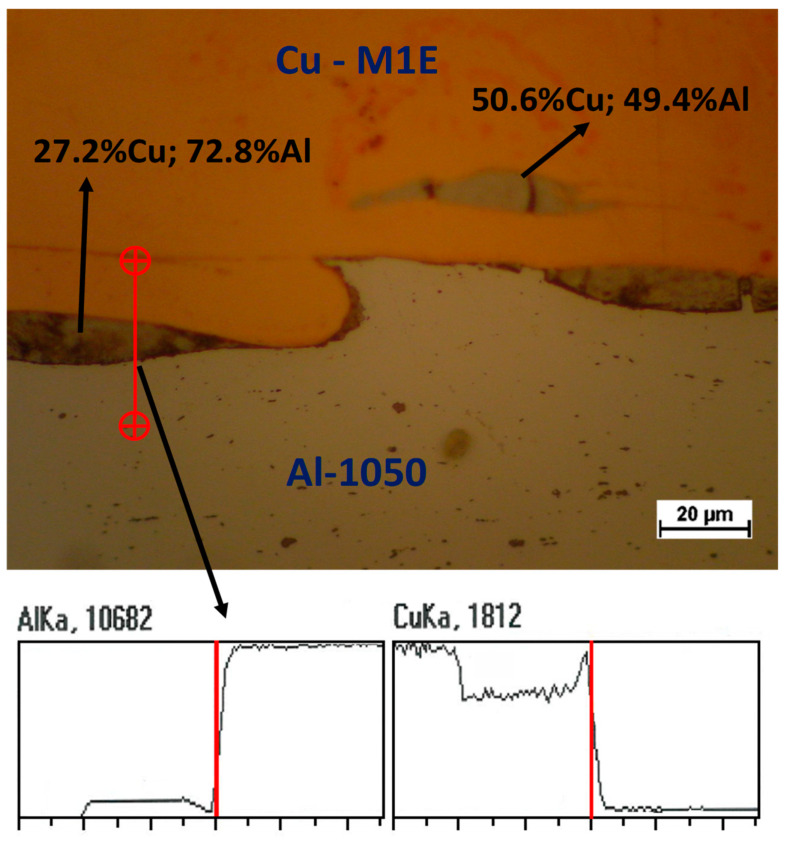
Example microstructure of the CuAl_2_ phase, obtained as a result of heat treatment, with markedly different measured chemical composition.

The presented example of the implementation of Al/Cu bimetal refers to the condition where copper is the outer layer in the finished product. As mentioned above, a problem that occurs very often in the manufacturing process of multilayer products, is the occurrence of intermetallic phases [7,9,10,11,12,13,14,15,16,17,18,19,20,25,27,29,30,31,32,33,34]. In bimetals of the Cu/Al type, a very commonly observed phase is CuAl_2_ (gray-brown area in Figure 3). This phase, which occurs in various varieties—e.g., Θ or η1—is very hard, which has been described by many authors [9,10,11]. It has a destructive effect on a bimetallic connection area. For many years, the authors of this article have been working with the issue of multilayer materials, for example bars and plates [17,23,24,34]. Example of papers concerning many materials such as two different steel grades [23] or Mg/Al [24] and Al/Cu [17,18]. In their research [10,18,24,34], the intermetallic phases were often observed. The main problem during deformation of multilayer materials in which there is a hard, brittle intermetallic phase is the presence of cracks in the merging area. This has been presented in the authors’ works [10,17,24,34]. Figure 3 shows an example of a microstructure and an exemplary chemical composition in various areas of these phases were obtained as a result of heat treatment. The presented example of the authors’ own work was obtained after stress–relief annealing in 315 °C for 30 min. Presented below. the red line indicates the scan direction and curves changes in Al and Cu content. The main advantage of presented in this work technology, is that use of explosive-welding with combination of cold ASR—without heat treatment operation—guarantees obtaining material with enough plasticity reserve to perform the drawing and pumping process.

## 2. Material Selection and Scope of the Research

**Figure 4:**
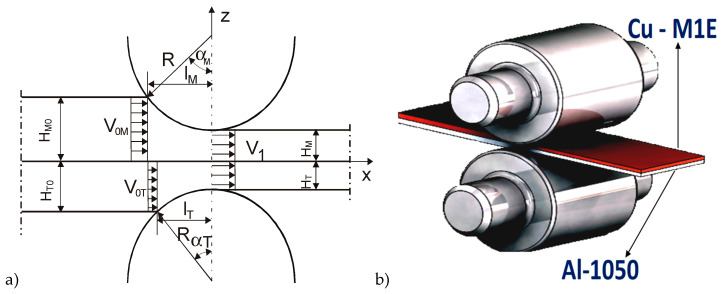
Asymmetrical rolling process of Al-1050 + Cu-M1E bimetallic sheets. (**a**) scheme of rolling process of bimetallic sheet with marked characteristic values, (**b**) schematic view of bimetallic sheet rolling process. Based on own study [7,8,9].

In the study, the multi-operational drawing process of Al-1050 + Cu-M1E bimetallic sheets obtained after explosion welding method initial connection and an asymmetrical rolling process was analyzed (Figure 4). The explosive-welding process generates a lot of heat, but its duration is short. The dynamics of the process prevented recording of temperature changes.

The values presented in the scheme of rolling process of bimetallic sheet, (Figure 4), means:

- H_M0_ and H_M_—the height before and after deformation of soft layer;
- H_T0_ and H_T_—the height of the hard layer;
- V_0 M_ and V_0 T_—the velocity of soft and hard layer.

In this study, the hard layer is the Cu-M1E and the soft layer is the Al-1050. Of course, the presented scheme is given only to facilitate understanding of the phenomena occurring during rolling of the multilayer materials. The chemical composition of the tested materials is presented in Table 1.

As part of the work, the Al/Cu deep-drawing process of two variants was analyzed: when the outer layer is copper, and when the outer layer is aluminum. Laboratory tests were carried out on discs cut out of bimetallic sheets with a diameter of 70 mm and a thickness of 1 mm. In the first stage the bimetallic sheets from which the samples were cut were asymmetrically rolled from an explosive-welded charge. The thickness of rolled bimetallic charge layers was: Cu-M1E—2 mm and Al-1050—10 mm. The process of the asymmetric rolling of bimetallic sheets was conducted in 8 passes. Due to the differences in the deformation resistances of the materials constituting the bimetal, the thickness share of the Cu-M1E layer in the rolled bimetal band increased from H_M1/HAl-1050_ = 0.17 to H_M1/HAl-1050_ = 0.25.

As a result, the materials constituting the bimetallic samples subjected to the drawing process had thicknesses, respectively, of: Cu-M1E = 0.2 mm and Al-1050 = 0.8 mm. For the first stage of research, a 34.6 mm diameter die, and a 32 mm diameter punch were used.

**Figure 5:**
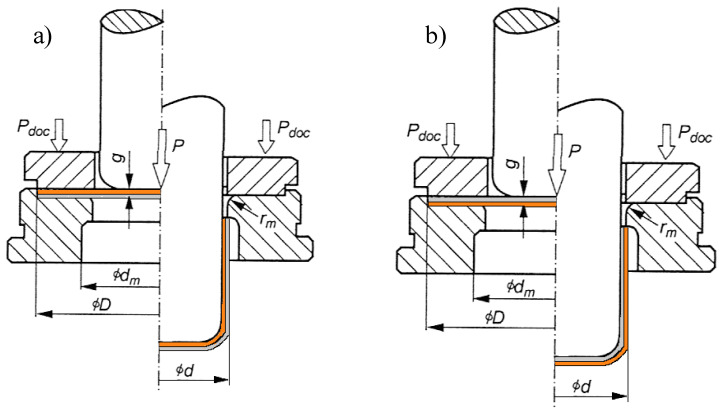
Conditions of the drawing process: (**a**) in the matrix–aluminum system (Al-1050 Cu-M1E), and (**b**) in the matrix–copper system (Cu-M1E Al-1050), left part in Figures before and right after drawing.

A drawing test was carried out with the coefficient m_1_ = 0.49 and two pressing operations with the coefficients m_2_ = 0.76 and m_3_ = 0.8, respectively. The pressing was carried out in two variants of bimetal contact with tools in the systems: matrix–Al-1050 (Figure 5a) and matrix–Cu-M1E (Figure 5b) [5,6].

In Figure 5, the arrows showing P_doc_ indicate the force with which the material was pressed. The arrow with the marked P indicates the piston force movement as a result of the impact of the pressing force on the material. The maximum value of this force was measured and shown in Figure 8. On the left side in Figure 5a,b the initial state before the drawing process is shown, while on the right the view after the process.

During the research, both the possibility of plastic forming of welded materials, as well as the quality and durability of the connection area of materials were analyzed. The aim of the research was also to determine the optimal conditions for plastic forming of thin bimetallic sheets after the rolling process.

## 3. Research Results and Their Analysis

**Figure 6:**
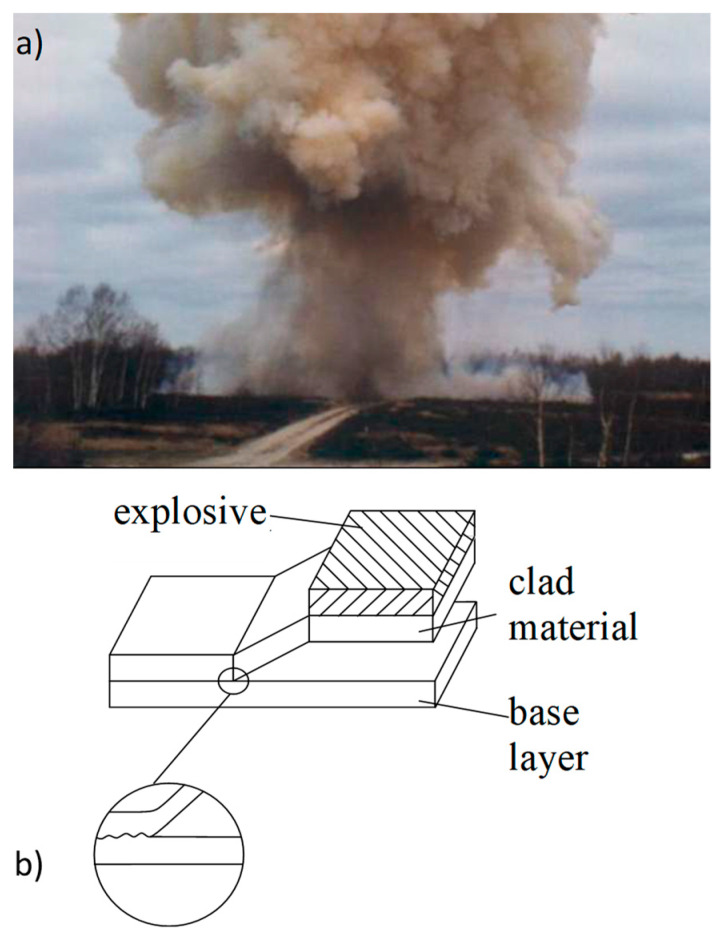
Explosive-welding of bimetallic sheets (**a**), schematic view of the process (**b**).

**Figure 7:**
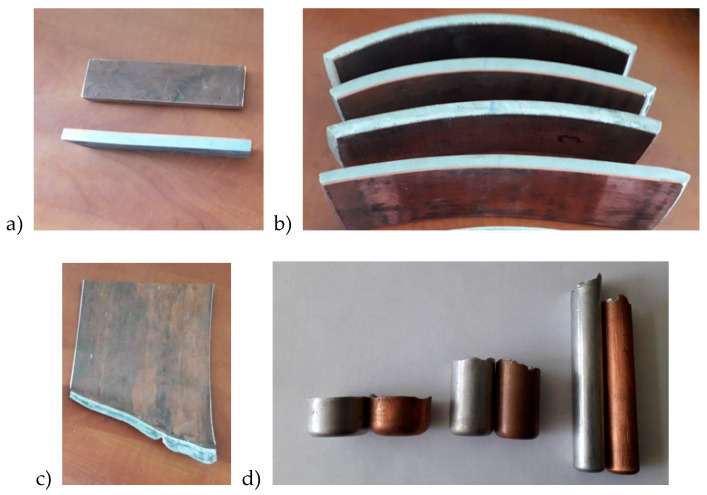
View of samples after subsequent stages of the technological process; (**a**) samples prepared for rolling; (**b**) sample after the last pass of the rolling process; (**c**) final samples after the rolling process; (**d**) samples after the drawing and pressing stages.

The study presents all stages of production, from explosive-welding, through preforming—asymmetrical rolling, to drawing. During the research it was found that layers position during deformation had a large impact on the value of force during drawing of double-layer materials. Figure 6 present the explosive-welding process. Figure 7 presents the bimetallic samples used for testing after subsequent stages of shaping. Figure 7a shows samples subjected to asymmetric rolling; Figure 7b shows a sample after the last pass of the rolling process; Figure 7c shows final samples after the rolling process and Figure 7d shows a view of the samples after drawing. As can be seen in Figure 7b, samples for subsequent passes of the rolling process were characterized by a certain curvature, depending on the parameters of asymmetrical rolling. Due to the ASR, it was possible to obtain an almost-straight sample which was used for further research stages.

The composites were obtained by means of a constant stand-off explosive-cladding technique. A detonator was placed in the middle of the plate, close to one of the edges.

The cold-rolling process was carried out on a D150-mm duo laboratory rolling mill. The aim of this stage of research was to obtain a proper material thickness for the pressing process. The speed of the upper roll was constant (100 mm/s), while the speed of the lower roll was variable—and dependent on the curvature of rolled bimetallic sheets. The rolling method depended on feeding samples to the rolling gap; the higher peripheral speed of the roller was from the harder layer. Asymmetrical rolling was performed with the use of roll-circumferential asymmetry a_v_ = V_lower_/V_upper_, which enabled smooth control of the plastic flow of the metal layers. The process was carried out for asymmetry coefficients a_v_ = 1.0–0.7. The rolling process was conducted in 8 passes. The initial dimensions of the rolled samples were H_0_ = 12 mm, B_0_ = 100 mm and L_0_ = 200 mm. The rolling direction was in-line with the direction of the explosion-wave propagation during explosion-welding. An individual optimal value of the asymmetry coefficient was determined for each pass, for which a flat, bimetallic sheet was obtained. Each time the same rotational speed of the working rolls was used (symmetrical rolling), curved samples were obtained. The value of the curvatures depended on the parameters of the rolling process. Table 2 presents the results of measurements for the rolling process of Al–Cu bimetallic sheets.

**Figure 8:**
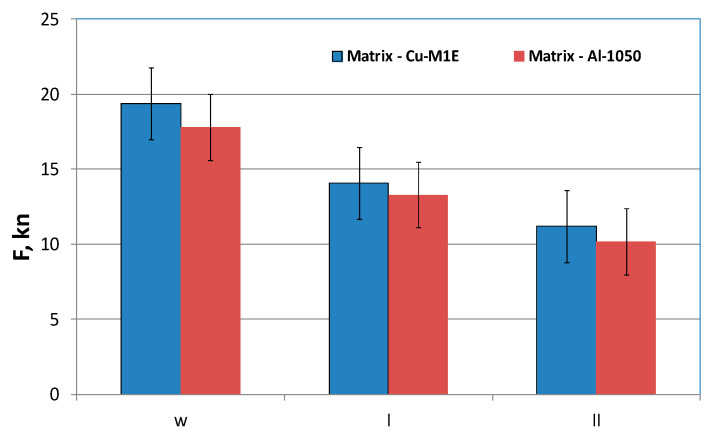
Registered values of maximum forces during the drawing (w) and pressing (I and II) processes.

The considerations for the drawing and pressing process of bimetallic samples after the rolling process are presented later in the work. In Figure 7d, a slight folded flange is visible after the pressing process. This problem appeared mainly under the influence of compressive peripheral stresses. This phenomenon increased in the subsequent stages of plastic forming and was often encountered during the extrusion or pressing process of thin sheets. During this research, an additional factor was observed the drawing process layers usually deform unevenly, and this fact increases the bearing. The unevenly deformed layers are clearly visible in Figure 7c,d—in the upper part of the sample. In the case of pressing of thin bimetallic sheets, this phenomenon is difficult or even impossible to control. It is particularly evident during a significant increase in the wall height obtained while reducing its thickness. An analysis of pressing forces was also made in this work; results are shown in Figure 8.

**Figure 9:**
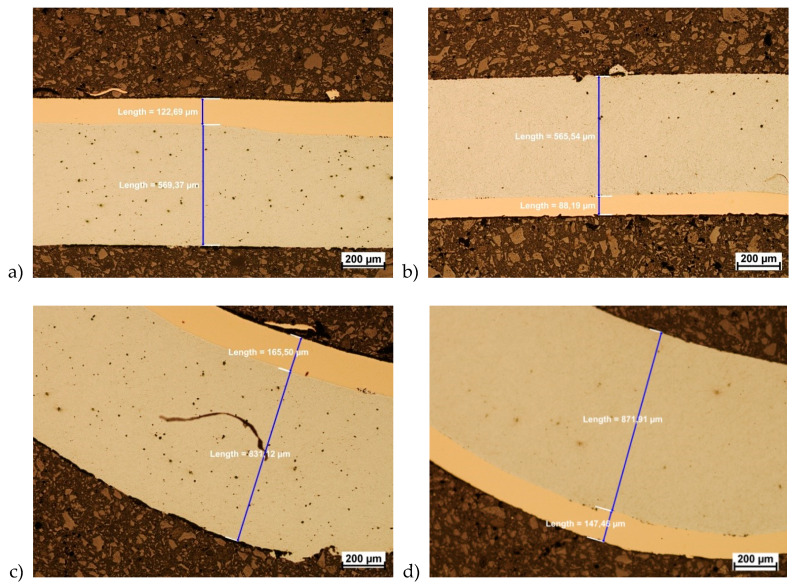
Welding area after the last drawing process. (**a**) side-wall area matrix–aluminum variant; (**b**) side-wall area matrix–copper variant; (**c**) matrix–aluminum variant in the sample rounding, (**d**) matrix–copper variant in the sample rounding.

**Figure 10:**
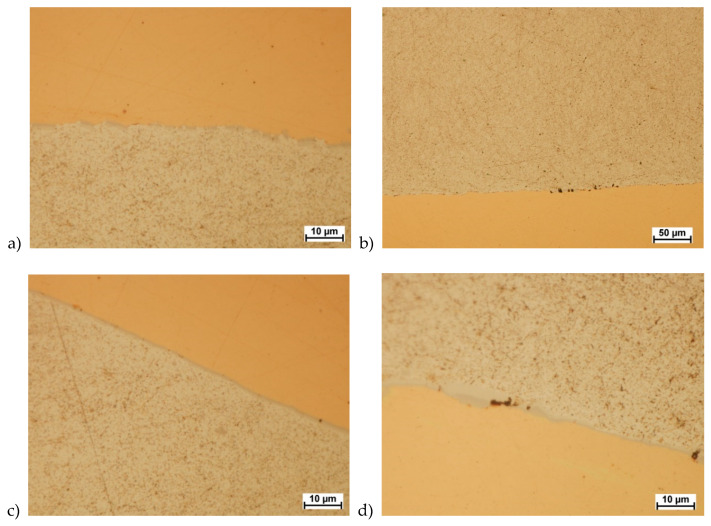
Welding area after the last pressing process. (**a**) side-wall area matrix–aluminum variant; (**b**) side-wall area matrix–copper variant; (**c**) matrix–aluminum variant in the rounding of the sample; (**d**) matrix–copper variant in the rounding of the sample.

Based on the results and values from carried-out tests presented in Figure 8, for two analyzed variants of material arrangement in drawing and pressing processes, it was observed that higher maximum-force values occur when the outer layer is Cu-M1E. The tested materials showed very good drawability. In order to assess the quality of the joint and changes in layer thickness, a microscopic evaluation was performed using optical microscopy. The analysis was carried out on non-etched samples. As a result, it was found that no delamination was observed in the connection area. During the drawing and pressing process, no cracks or delamination were observed in the bimetallic welding zone (Figure 9 and Figure 10). The analysis was conducted in the two areas that were considered as critical, i.e., the area of strongest stretching (the side of the part) and the area of bending at the bottom. Figure 9 shows a microscopic view of both zones of individual variants of layer arrangement during drawing.

Based on the microscopic analysis, slight differences in layer thicknesses were found (Figure 9). The differences were observed in the height of samples. These differences are obvious and are related to the materials plasticity. A greater reduction in the thickness of the Cu-M1E layer occurred where it contacted the matrix. This knowledge was needed from the technological point of view because areas of lower wall thickness are more vulnerable to damage. Interestingly, the thickness of the layers in all three analyzed areas for both variants were similar. Table 3 presents the average values of the changes in the layer thicknesses.

Microscopic analysis also showed that the welding area was almost free of intermetallic phases. Only small individual precipitations were observed (Figure 10d). This is especially important because hard and brittle layers of the type CuAl_2_ cause delamination—especially during plastic forming processes. Sample observation results are shown in Figure 10.

**Figure 11:**
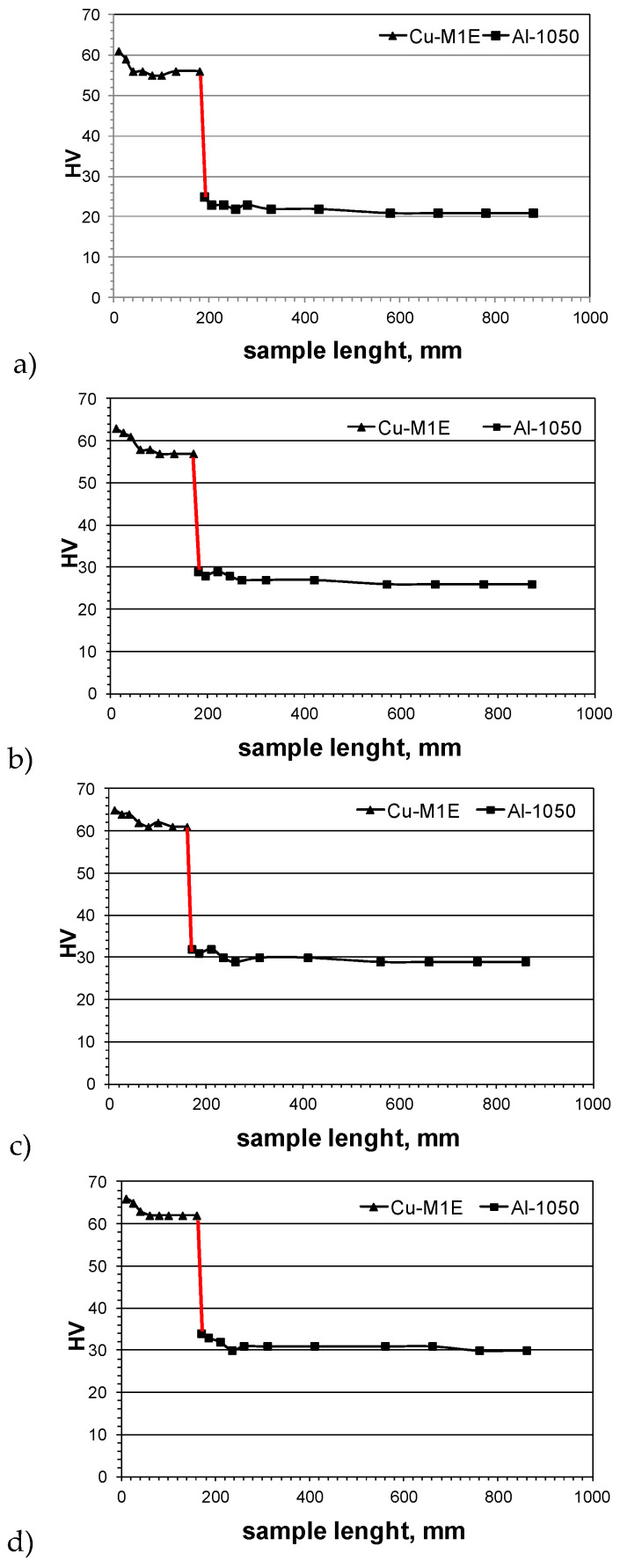
Results of hardness measurement in Al-1050 + Cu-M1E bimetal layers (in the rounding-area). (**a**) after the rolling process; (**b**) after the drawing process; (**c**) after the pressing process I; (**d**) after the pressing process II. Red line show the welding area.

As can be seen, not only was there almost no precipitation of intermetallic phases, but also the connection area was flat, which guaranteed good plasticity and formability. Next, microhardness tests were conducted. The aim of these tests was to show the effect of plastic-deformation on the mechanical properties of the bimetal materials during drawing and pressing. The microhardness measurement was carried out by Vickers method using a semi-automatic Future-Tech FM700 microhardness tester. The averaged results of the performed tests for samples after subsequent stages of plastic-forming of the Cu-M1E + Al-1050 bimetal are shown in Figure 11.

Based on the results of the microhardness tests presented in Figure 11, it was found that the hardness of individual layers was the greatest in the area of the joint. With the increase of distance, it slightly decreased. The greatest differences in layer hardness were observed after the rolling process. However, it should be mentioned that after drawing and pressing, values for both were similar. This confirmed that the material has a very good formability and a reserve of plasticity.

## 4. Summary

In the current work, bimetallic materials pre-connected by methods of explosive-welding were analyzed. The materials were subjected to asymmetric rolling, drawing and pressing processes. On the basis of our research and the results of the plastic formation process of thin bimetallic sheets Cu-M1E + Al-1050 after the cold-rolling process, it can be concluded that this process is possible. No delamination or cracks were observed in the area of connection. The plastic forming-process was carried out without heat treatment, which caused formation of intermetallic phases. The lack of heat treatment allowed avoiding the formation of microcracks and cracks—and as a result—did not leads to delamination of the joined materials. The presented results indicate that it is possible to obtain a finished, explosively welded, pre-rolled and pressed Cu-M1E + Al-1050 bimetallic product. The considerations carried out in this work demonstrate that as a result of controlled plastic deformation processes, optimal conditions for their implementation can be developed. The results obtained in this work can and should be used as guidelines for the development of comprehensive technologies for plastic forming of bimetals from non-ferrous materials in industrial conditions.

## Figures and Tables

**Table 1:** Chemical composition of tested materials (mass percent).

Material	Cu	Al	Fe	Si	Zn	Ti	Mg	Mn
M1E	99.9	–	max 0.005	max 0.002	max 0.003	max 0.002	–	–
Al-1050	max 0.05	99.5	max 0.4	max 0.25	max 0.07	max 0.05	max 0.05	max 0.05

**Table 2:** Results and parameters for asymmetrical rolling process.

Pass No	Total Thickness, mm	Total Deformation %	Layer Thickness Cu, mm	Layer Thickness Al, mm	Layer Thickness Ratio gCu/gAl	Asymmetry Coefficient av
0	12.00	-	2.00	10.00	0.20	-
1	10.10	15.83	1.81	8.29	0.22	0.92
2	8.50	29.70	1.61	6.89	0.23	0.91
3	6.90	42.50	1.33	5.57	0.24	0.89
4	5.05	57.92	0.98	4.07	0.24	0.80
5	3.80	68.33	0.72	3.08	0.24	0.78
6	2.70	77.50	0.53	2.17	0.24	0.80
7	2.00	83.33	0.40	1.60	0.25	0.79
8	1.30	89.17	0.26	1.04	0.25	0.73
9	1.00	91.67	0.20	0.80	0.25	0.80

**Table 3:** Results of measurements of average values of layer thickness for the examined processes.

Variant	Place of Measurement	Variant Matrix–Al1050-M1E		Variant Matrix–M1E-Al1050	
		Thickness Al1050, mm	Thickness Cu-M1E, mm	Thickness Al1050, mm	Thickness Cu-M1E, mm
Drawing	Top	0.82	0.18	0.82	0.18
	Rounding	0.81	0.19	0.81	0.19
	Bottom	0.82	0.18	0.82	0.18
Pressing I	Top	0.64	0.16	0.65	0.15
	Rounding	0.81	0.18	0.81	0.17
	Bottom	0.82	0.18	0.81	0.19
Pressing II	Top	0.40	0.10	0.40	0.10
	Rounding	0.81	0.18	0.8	0.17
	Bottom	0.78	0.19	0.78	0.18
